# Individual and Combined Impact of Oxygen and Oxygen Transporter Supplementation during Kidney Machine Preservation in a Porcine Preclinical Kidney Transplantation Model

**DOI:** 10.3390/ijms20081992

**Published:** 2019-04-23

**Authors:** Abdelsalam Kasil, Sebastien Giraud, Pierre Couturier, Akbar Amiri, Jerome Danion, Gianluca Donatini, Xavier Matillon, Thierry Hauet, Lionel Badet

**Affiliations:** 1 INSERM U1082 (IRTOMIT), Poitiers F-86000, France; akasil2011@gmail.com (A.K.); giraudseb@yahoo.fr (S.G.); pierre.couturier@outlook.fr (P.C.); akbar_amiri@hotmail.com (A.A.); jerome.danion@me.com (J.D.); gianluca.donatini@chu-poitiers.fr (G.D.); xav.matillon@gmail.com (X.M.); lionel.badet@chu-lyon.fr (L.B.); 2 Faculté de Médecine et Pharmacie, Université de Poitiers, Poitiers F-86000, France; 3 Service d’Urologie et de Chirurgie de la Transplantation, Hôpital Edouard Herriot, Lyon F-69003, France; 4 Service de Biochimie, CHU Poitiers, Poitiers F-86000, France; 5 Plate-forme Infrastrutures en Biologie Sante et Agronomie (IBiSA) MOdélisation Préclinique - Innovations Chirurgicale et Technologique (MOPICT), Domaine Expérimental du Magneraud, Surgères F-17700, France; 6 Service de Chirurgie viscérale, CHU de Poitiers, Poitiers F-86000, France; 7 Université Claude Bernard Lyon 1, Lyon F-69003, France; 8 CarMeN Laboratory, INSERM U1060, Villeurbanne F-69100, France; 9 FHU SUPORT ‘SUrvival oPtimization in ORgan Transplantation’, Poitiers F-86000, France

**Keywords:** dynamic/machine perfusion, oxygen, hemoglobin, ischemia, kidney transplantation

## Abstract

Marginal kidney graft preservation in machine perfusion (MP) is well-established. However, this method requires improvement in order to mitigate oxidative stress during ischemia-reperfusion, by using oxygenation or an O_2_ carrier with anti-oxidant capacities (hemoglobin of the marine worm; M101). In our preclinical porcine (pig related) model, kidneys were submitted to 1h-warm ischemia, followed by 23 h hypothermic preservation in Waves^®^ MP before auto-transplantation. Four groups were studied: W (MP without 100%-O2), W-O_2_ (MP with 100%-O2; also called hyperoxia), W-M101 (MP without 100%-O2 + M101 2 g/L), W-O_2_ + M101 (MP with 100%-O2 + M101 2 g/L) (*n* = 6/group). Results: Kidneys preserved in the W-M101 group showed lower resistance, compared to our W group. During the first week post-transplantation, W-O_2_ and W-M101 groups showed a lower blood creatinine and better glomerular filtration rate. KIM-1 and IL-18 blood levels were lower in the W-M101 group, while blood levels of AST and NGAL were lower in groups with 100% O2. Three months after transplantation, fractional excretion of sodium and the proteinuria/creatinuria ratio remained higher in the W group, creatininemia was lower in the W-M101 group, and kidney fibrosis was lower in M101 groups. We concluded that supplementation with M101 associated with or without 100% O2 improved the Waves^®^ MP effect upon kidney recovery and late graft outcome.

## 1. Introduction

Kidney transplantation is the best treatment for patients with end-stage kidney disease, but unfortunately, the current organ shortage is a major challenge. This shortage leads to increased acceptance of high risk donors, such as Donation after Circulatory Death (DCD) and expanded criteria donors (ECD). Kidneys procured from such donors are more sensitive to ischemia-reperfusion (IR), and subsequently more susceptible to tardive lesions/dysfunctions [1]. To decrease these marginal donor-related complications, graft preservation conditions need to be optimized. Despite the demonstrated benefits of machine perfusion (MP) in preserving DCD kidneys, the complications arising from DCD graft transplantation remain elevated [2]. Hence, this MP method still requires improvement to minimize ischemia-related injuries.

Previous studies have reported the interest of oxygenation during MP [3,4]. Otherwise, the medical device HEMO_2_life^®^ (HEMARINA, Morlaix, France) contains a natural extracellular hemoglobin (M101), isolated from the marine lugworm (*Arenicola marina*). This biopolymer of high molecular weight (3600 kDa) has a large O_2_ binding capacity, carrying up to 156 O_2_ molecules. It releases O_2_ according to a simple gradient, without requiring any allosteric effector [5]. In addition, it was demonstrated that M101 has an intrinsic Cu/Zn-Superoxide dismutase (SOD) antioxidant activity [6], a highly valuable property of in the context of IR, where a major production of reactive oxygen species (ROS) is known to occur.

This medical device has recently been developed for organ protection in transplantation [6]. It was demonstrated that M101 added to the kidney preservation solution considerably reduced the delayed graft function (DGF) and tardive lesions (as fibrosis) in an in vivo porcine model of autotransplantation [6], results recently confirmed in a multicentric human clinical trial (OxyOp; NCT02652520) [7]. Nevertheless, the M101 supplementation in a preservation solution was never evaluated in any oxygenated kidney perfusion machine before transplantation.

Although there is consensus on the MP benefits for DCD kidneys, there is not yet clear for using oxygenation perfusion [3,4] however, despite the fact that studies have demonstrated the benefits of oxygenated perfusion [8]. The new transportable Waves^®^ MP system provides controlled pulsatile kidney perfusion with the possibility of connecting the device to an external gas source (such as an oxygen bottle) to generate an oxygenated hypothermic perfusion condition. This new MP also allows the use of different oxygen levels during perfusion, offering flexibility compared to other transportable MP devices. This potential for oxygenated hypothermic machine perfusion provided by the Waves^®^ device could be an interesting way of supplying oxygen to the organ. In addition, because oxygen could be a toxic component, and can provoke oxidative damage if not physiologically delivered, such technical protocol is of paramount interest for an adequate clinical use. Thus, use of an oxygen carrier with antioxidant capacities, as M101, could further optimize preservation quality and regulate toxic hyperoxia.

Therefore the right level for the oxygen supply and delivery modalities during cold preservation remains to be specified. In this study, we have sought to assess the effects of hyperoxia (100% O_2_, also called hyperoxia) ± M101 supplementation (respectively or combined) during ex vivo kidney perfusion in a preclinical 3-month follow-up pig kidney autotransplantation model. This study’s primary endpoints were post-transplantation renal function recovery and fibrosis development at 3 months. In clinics, the kidneys from DCD are exposed to a warm ischemia (WI) period (no-flow after cardiac arrest) prior to kidney harvesting and cold ischemia. Thus, herein we used an experimental model mimicking the DCD situation, with 1 h WI prior to cold ischemia in a perfusion machine. Indeed, 1 h WI has been established in our laboratory as the best compromise between tissue injury and animal survival [3,9,10].

In this present study, we used M101 at 2 g/L, since we have recently shown, in the above DCD-like model, that kidney preservation in a machine perfusion with this concentration (as compared to 0 g/L and 1 g/L M101) translated into higher benefits for graft function recovery [11].

## 2. Results

### 2.1. Ex Vivo Perfusion Parameters Evaluation

**Figure 1:**
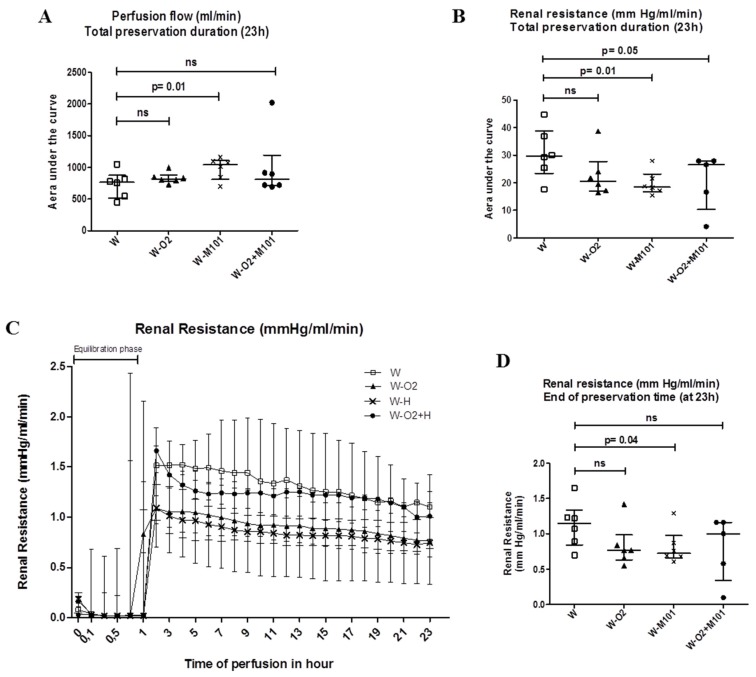
Ex vivo perfusion parameters. Area under the curve (AUC) representations of perfusion flow (**A**) and renal resistance during 23 h of cold preservation in machine perfusion (MP) Waves^®^ (**B**). Time evolution of renal resistance during 23 h of cold preservation in MP Waves^®^ (**C**) and at end of preservation time (**D**). Of note, the initial, "no resistance" phase (first hour, panel **C**) corresponds to the set-up and temperature equilibration phase of the machine (no kidney graft mounted). Results are expressed as median and interquartile range (IQR); statistical analysis was performed with Kruskal-Wallis Multiple comparison Dunn’s test. *n* = 5–6/group.

There was a slight increase in the perfusion flow in groups with M101 supplementation (W-M101 and W-O_2_ + M101), compared to the groups without M101 (W and W-O_2_), with significant differences between group W versus group W-M101 (*p* = 0.01) (Figure 1A). Both groups with M101 showed less renal vascular resistance, with a significant difference between W-M101 versus W groups, and W-O_2_ + M101 versus W (*p* = 0.01 and *p* = 0.05, respectively; Figure 1B,C). This was confirmed by better end-perfusion resistance between W-M101 versus W (*p* = 0.04) (Figure 1D).

### 2.2. Kidney Function Recovery from Day 0 to Day 7 Post-Transplantation

**Figure 2:**
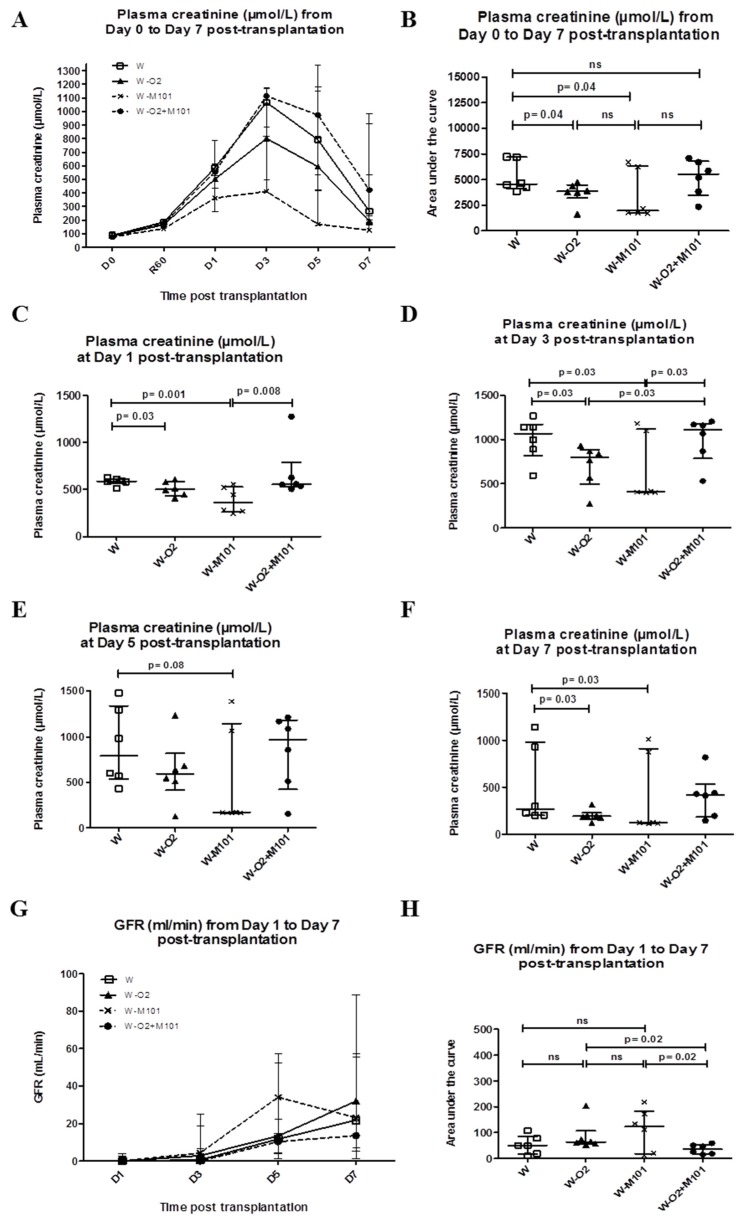
Evaluation of early kidney function recovery from day 0 to day 7 post-transplantation. Evolution of plasma creatinine level (**A**–**F**) and glomerular filtration rate (GFR) (**G**–**H**) in the different experimental groups during the first week of post-transplantation (Day 0 to Day 7). Results are expressed in the kinetic curve (**A**,**G**) and AUC (**B**,**H**). Results are expressed in median with interquartile range (IQR). Statistical analysis was performed with Kruskal-Wallis Multiple comparison Dunn’s test. *n* = 6/group.

All animals survived after kidney transplantation. In term**s** of urine production recovery, at day 1 post-transplantation, 33.3% of animals had urine production in W + O_2_ or/and M101 groups, compared to 16.6% in the W group. At day 2 post-transplantation, 66.6% of the animals had urine production in W, W-O_2_ and W-M101 groups, compared to 50% in group W-O_2_ + M101. All animals had positive diuresis at day 3 post-transplantation. From day 0 to day 7 post-transplantation, plasma creatinine peaked at day 3 in all groups (Figure 2A). 

As regards benefits induced by M101 and O_2_, plasma creatinine AUC analysis shows significant differences between W-M101 versus W (*p* = 0.04; Figure 2B). Although, creatinine level was lower in the W-M101 group compared to the W and W-O_2_ + M101 groups at days 1, 3, 5 and 7 post-transplantation (Figure 2C–F). Glomerular filtration rate (GFR) analysis showed that W-M101 and W-O_2_ groups were significantly better, compared to the W-O_2_ + M101 group (*p* = 0.04) (Figure 2G–H) (GFR normal value at day 0 = 20–40 mL/min, day 7 = 30–50 mL/min and AUC Day 0–7 = 175 mL/min). GFR levels were not different between groups at any time during the first week post-transplantation, and neither was the Fractional Excretion of Sodium (FeNa; data not shown).

### 2.3. Kidney Injury Biomarker Evaluation from Day 0 to Day 7 Post-Transplantation

**Figure 3:**
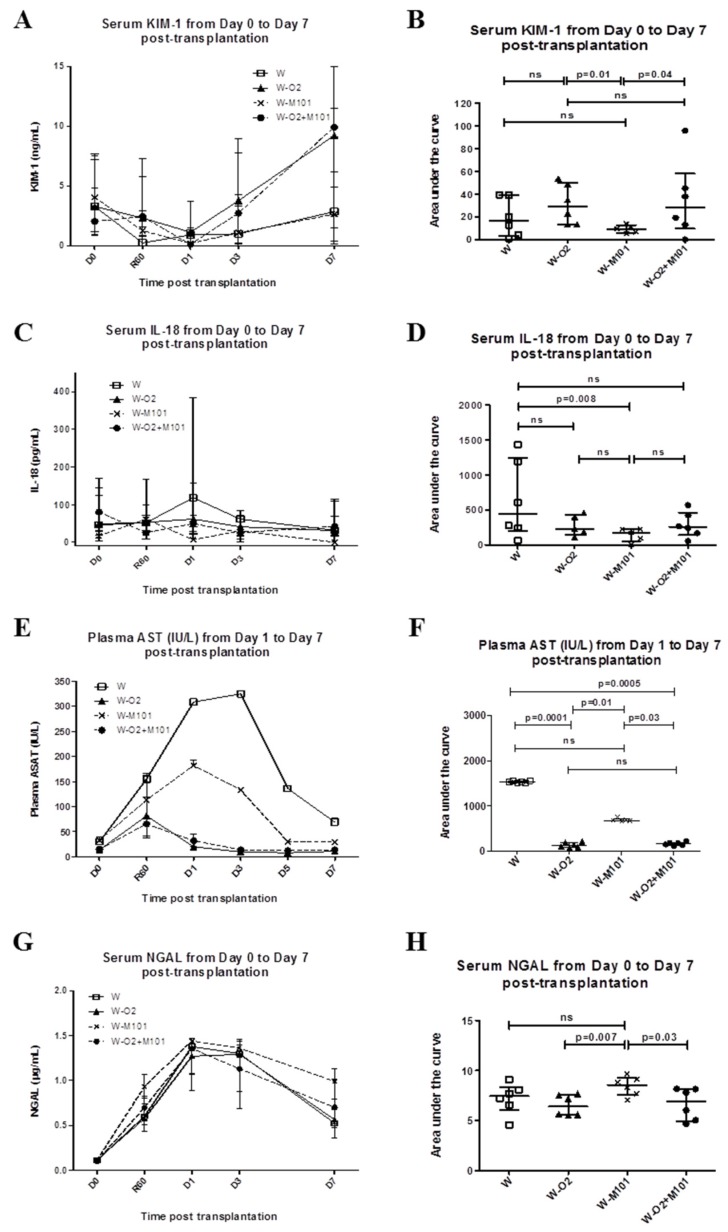
Evaluation of kidney injury biomarkers from day 0 to day 7 post-transplantation. Evolution of serum KIM-1 level (**A**,**B**), IL-18 (**C**,**D**), plasma AST (**E**,**F**) and NGAL (**G**,**H**) in the different experimental groups during the first week of post-transplantation (Day 0 to Day 7). Results are expressed in the kinetic curve (**A**,**C**,**E**,**G**) and area under the curve (**B**,**D**,**F**,**H**). Results are expressed in median with interquartile range (IQR). Statistical analysis was performed with Kruskal-Wallis Multiple comparison Dunn’s test. *n* = 5–6/group.

Blood 8-isoprostane (reflect of ROS) was not detected in any group in the first 3 days post-transplantation. Because urine production was inconstant post-transplantation, serum levels of injury biomarkers KIM-1, IL-18, NGAL and plasma AST were analyzed over the first week. The groups without 100% O_2_ (W and W-M101) showed lower KIM-1 peak levels (~2.5–3 ng/mL) than the groups with 100% O_2_ (~9–10 ng/mL) (Figure 3A). AUC analysis showed a significant difference between the W-M101 group versus the W-O_2_ group and the W-M101 group versus the W-O_2_ + M101 group (*p* = 0.01 and *p* = 0.04 respectively; Figure 3B). The groups without M101 supplementation revealed higher levels of serum IL-18 at day 1 (118 pg/mL) for the W group and (62 pg/mL) for the W-O_2_ group in comparison to the groups with M101 supplementation (6 pg/mL) for the W-M101 group and (50 pg/mL) for the W-O_2_ + M101 group (Figure 3C). AUC analysis showed significant IL-18 reduction with M101 supplementation in the groups without 100% O_2_ (W-M101 versus W) (*p* = 0.008) (Figure 3D). The groups without 100% O_2_ (W and W-M101) showed high plasma AST (~200–400 IU/L) compared to the groups with 100% O_2_ (W-O_2_ and W-O_2_ + M101) (~60–80 IU/L). This difference was statistically significant in the groups without 100% O_2_ versus other groups: W versus W-O_2_ (*p* = 0.0001), W versus W-O_2_ + M101 (*p* = 0.005), W-M101 versus W-O_2_ (*p* = 0.01) and W-M101 versus W-O_2_ + M101 (*p* = 0.03) (Figure 3E–F). All the groups showed positive levels of the serum NGAL. The group with 100% O_2_ (W-M101) showed high serum NGAL (~0.8µg/mL) compared to the groups with 100% O_2_ (W-O_2_ and W-O_2_ + M101) (~0.6 µg/mL). This difference was statistically significant in the group without 100% O_2_ versus the other groups: W-M101 versus W-O_2_ (*p* = 0.007), W-M101 versus W-O_2_ + M101 (*p* = 0.03) (Figure 3G–H).

### 2.4. Kidney Function and Injury Biomarker Evaluation at Day 14 Post-Transplantation

**Figure 4:**
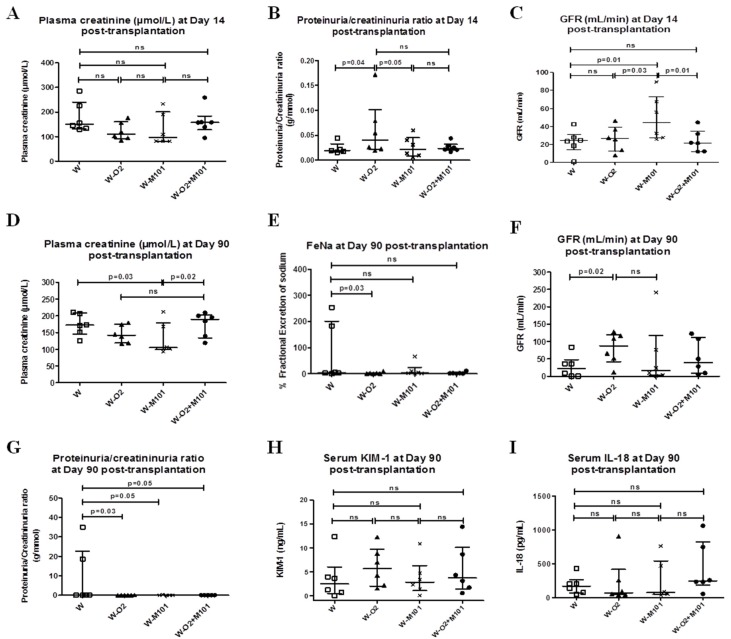
Evaluation of kidney function and injury biomarkers at day 14 and 90 post-transplantation. Evolution of plasma creatinine level (**A**), proteinuria/creatinuria ratio (**B**) and glomerular filtration rate (GFR) (**C**) at day 14 of post-transplantation. Evolution of plasma creatinine level (**D**), fractional excretion of sodium (FeNa) (**E**), glomerular filtration rate (GFR) (**F**), proteinuria/creatinuria ratio (**G**), serum KIM-1 (**H**) and IL-18 (**I**) at day 90 post-transplantation. Results are expressed in median with interquartile range (IQR). Statistical analysis was performed with Kruskal-Wallis Multiple comparison Dunn’s test. *n* = 5–6/group.

At day 14 post-transplantation, plasma creatinine was lower in groups W-O_2_ (111 µmol/L) and W-M101 (95.5 µmol/L) in comparison to the groups W (155 µmol/L) and W-O_2_ + M101 (157.5 µmol/L), but without significant difference between the groups (Figure 4A). The proteinuria/creatinuria ratio (sign of renal damage) was higher in group W-O_2_ versus the groups W and W-M101 (*p* = 0.04 and *p* = 0.05 respectively, Figure 4B). 

The group W-M101 had better GFR than other groups; W-M101 versus W (*p* = 0.01), W-M101 versus W-O_2_ (*p* = 0.03) and W-M101 versus W-O_2_ + M101 (*p* = 0.01) (Figure 4C) (GFR normal value at day 14 = 55 mL/min).

### 2.5. Kidney Function Evaluation at Day 90 Post-Transplantation

After 90 days of follow-up, we observed that plasma creatinine of W-M101 group animals was lower compared to W (*p* = 0.03) and W-O_2_ + M101 (*p* = 0.02) (Figure 4D). The group W had more FeNa (4%) than other groups (W versus W-O_2_, *p* = 0.03) (Figure 4E). The group W-O_2_ had significantly better GFR compared to the W group (*p* = 0.02) (Figure 4F) (GFR normal value at day 90 = 80 mL/min). The proteinuria/creatinuria ratio was significantly higher in the group W versus W-O_2_ (*p* = 0.03), versus W-M101 (*p* = 0.05) and versus W-O_2_ + M101 (*p* = 0.05) (Figure 4G). At day 90 post-transplantation, levels of serum KIM-1 and IL-18 were not significantly different between groups, highlighting the exclusively early interest of these biomarkers after renal injury (Figure 4H–I).

### 2.6. Histological Evaluation at Day 90 Post-Transplantation

**Figure 5:**
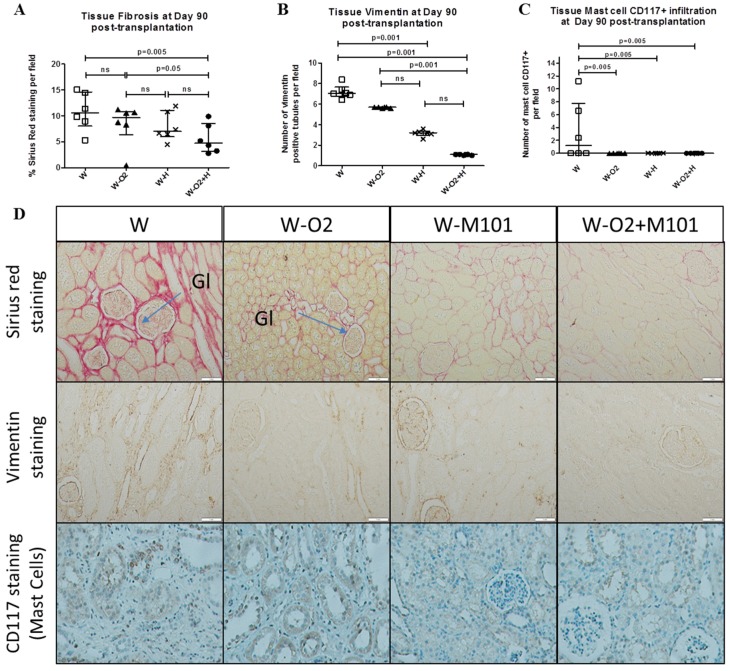
Evaluation of histological kidney graft tissue at day 90 post-transplantation. Quantification of tissue fibrosis (**A**; % of Sirius Red staining/field), number of vimentin positive tubules (**B**) and mast cells infiltration (**C**; CD117 staining) at day 90 post-transplantation, in histological sections obtained from renal kidney cortices of different groups. Related representative images for each group (**D**) showing Sirius Red staining, vimentin staining and CD117 positive mast cells staining in cortex kidney graft tissue at day 90 post-transplantation (magnification ×100). Results are expressed in median with interquartile range (IQR). Statistical analysis was performed with Kruskal-Wallis Multiple comparison Dunn’s test. *n* = 6/group.

Interstitial fibrosis evaluated by % of Sirius Red staining per field in transplanted kidneys was significantly higher in the groups without M101 supplementation, compared to the groups with O_2_ + M101 supplementation (*p* < 0.05) (Figure 5A). Similarly, evaluation of tissue kidney vimentin revealed significantly higher expression in the groups without M101 supplementation (W and W-O_2_), compared to the groups with M101 supplementation (W-M101 and W-O_2_ + M101) (*p* = 0.001) (Figure 5B). The renal tissue infiltration by leucocytes and particularly mast cell CD117^+^ was also significantly higher in W group, compared to all the other groups: (*p* = 0.005) (Figure 5C).

## 3. Discussion

Using a porcine kidney transplantation model, we evaluated the respective effects of hyperoxia (100% O_2_) or not with or without M101 supplementation during MP kidney preservation, on graft transplantation outcome.

It has been demonstrated that renal vascular resistance in hypothermic machine perfusion (HMP) is associated with graft recovery and survival [12,13]. In relation with these results, we evaluated the ex vivo HMP parameters. During HMP preservation, the W group (no supplementation) showed the worst results in terms of perfusion flow, and thus renal resistance. Interestingly, we observed that the M101 supplementation groups had lower renal resistance. This could be due to the anti-ROS activity of M101 (intrinsic SOD property) and the pO_2_ regulation capacities of this hemoglobin. In fact, M101 has a high affinity to oxygen (low P_50_), making it an oxygen carrier able to deliver oxygen specifically to hypoxic tissues [5].

We observed the benefits of M101 supplementation or 100% O_2_ alone on the plasma creatinine peak, which was lower between day 3 and day 7 than in other groups. These findings support previous studies showing that these supplementations would be effective regardless of the type of MP [3,6]. In addition, we noted the MP Waves^®^ benefits compared to static cold storage preservation in UW (creatinine peak of ~600 µmoL/L at day 14 post-transplantation *versus* ~1250 µmoL/L) [14].

We also evaluated the biomarkers KIM-1, IL-18, NGAL and AST after transplantation as indicators of renal injury. They were determined at the blood level instead of the urine level, because urine production after transplantation was not constant [15,16]. Blood evaluation is easier than in urine in clinical settings in the first days post-transplantation (in case of anuria), and in addition, several studies demonstrated that blood evaluation of these biomarkers is relevant and correlated to urine expression [15,16,17,18].

Plasma AST is an enzyme reported as a good biomarker of renal injury in a preclinical pig model of kidney transplantation [17]. We observed that 100% O_2_, M101 or combination groups exhibited less AST excretion, supporting the previously-reported hypothesis of a protective effect of oxygenation and oxygen carrier. Blood NGAL is produced by damaged kidney tubular cells and active neutrophils, and is predictive of transplant lesions [17]. In our study, serum NGAL levels were significantly lower in 100% O_2_ groups, suggesting that O_2_ could be beneficial in limiting tubular cell injury during extracorporeal perfusion. In addition, the M101 and 100% O_2_ combined condition reduced NGAL levels as compared to M101 alone.

KIM-1 is markedly upregulated in the proximal tubular epithelium after injury [15]. It is known that ischemic insults to the kidney cleave the cell surface KIM-1 ectodomain, the latter being detectable early after kidney injury [15]. It has been proposed that KIM-1 exposed on the apical surface of surviving proximal tubule epithelial cells plays an important role in kidney recovery, because it acts as a phosphatidylserine receptor, mediating efferocytosis to prevent tubular obstruction by apoptotic cells [19,20]. Otherwise, as confirmed in an ex vivo model, ROS enhances the cell surface KIM-1 shedding, and it was speculated that excess KIM-1 proteolysis might occur during severe AKI, and limit renal recovery [19,20]. Herein, an early high level of circulating KIM-1 was observed in the group of 100% O_2_ alone, and M101 alone appeared to act as a protective condition, reducing KIM-1 cleavage. However, the combined addition of M101 and 100% O_2_ showed high levels of circulating KIM-1, compared to the group M101 alone. This could be explained by the hyperoxia associated with the 100% O_2_ condition; for instance, the latter may have induced exacerbated ROS production and concomitant cell injury [4,21]. However, no difference was observed regarding KIM-1 excretion in the different experimental groups after 3 months.

As regards M101 anti-ROS activity, our data suggest that ROS/anti-ROS balance could be a major key to limit injury level, and that M101 could have a cardinal role [6,19]. Moreover, acute KIM-1 expression could be modulated, and plays an important role in modulation of the innate immune response in AKI [19,20]. These data suggest a protective role of KIM-1 on kidneys early after injury via processes facilitated by apoptotic cell uptake. In turn, tardive KIM-1 expression could be an indicator of a maladaptive situation leading to chronic kidney disease [20].

We also studied IL-18, a pro-inflammatory cytokine, produced by damaged proximal tubule cells, and increased early after kidney injury [16,18]. Soluble IL-18 is also used as a biomarker of disease progression because IL-18 contributes to the process of organ damage and has an important role in inflammation and fibrosis pathogenesis [18]. In our study, we noted that increased levels of serum IL-18 in the W group is associated with a decrease in estimated GFR, increases in the proteinuria/creatinuria ratio and plasma creatinine level, as well as fibrosis and leucocyte infiltration, corroborating previous reports [18,22]. IL-18 is an important mediator in ischemic kidney injury that promotes leukocyte infiltration to kidney parenchyma, and it is correlated with the severity of proteinuria [23]. In our model, IL-18 seems to be a valuable biomarker, because its secretion profile between the groups is associated with late complications (GFR, proteinuria/creatinuria ratio, fibrosis and leukocyte infiltration). Interestingly, the correlation between lower renal resistance (in HMP), better diuresis recovery, lower blood IL-18 (first week post-transplantation) and reduced fibrosis and creatinine levels at day 90, in the W-M101 group, suggests that the early increase in blood IL-18 level, associated with renal resistance, could be a predictive indicator of kidney outcome. Our data are supported by a previous report showing that increased renal resistance during HMP is associated with high incidences of DGF and survival graft [12,13,24]. Interestingly, in our hands, from day 0 to 7, blood KIM-1 and IL-18 were associated with M101 supplementation benefits, whereas NGAL and AST seemed to be associated with oxygenation benefits. Such results suggest that these biomarkers could help constitute a working algorithm to predict organ recovery. 

Three-months post-transplantation follow-up enabled us to estimate the extent of late interstitial fibrosis and tubular atrophy associated with chronic graft dysfunction and graft loss [25]. Herein, we noted the MP Waves^®^ benefits, as compared to static cold storage conservation in UW (~20% of fibrosisat day 90 post-transplantation) [14].

Kidneys preserved using MP with M101 supplementation showed significant less fibrosis than other groups without M101 (*p* < 0.05). Vimentin expression in renal tissues is an established marker of endothelial to mesenchymal transition (EMT) known to boost fibrosis [26]. We observed that vimentin expression was lower in both groups supplemented with M101. Also, using 100% O_2_ alone (i.e. without M101) in MP seems to be damageable to the kidney, with a tardive vimentin expression and increased Red Sirius staining. These data are consistent with previous reports focusing on the risks of high-flow oxygen during preservation [4]. In addition, M101 could reduce this side effect and limit high level oxygen toxicity and potential long term effects. It is well-known that mast-cell leucocyte infiltration increases in a damaged kidney parenchyma, and that such an infiltration is correlated to fibrosis level, via their tryptase secretion (which enhances the fibroblastic proliferation [27]). In our model, mast-cell leukocyte infiltration was high in the W group, while it was nearly absent in other groups.

In our model, perfusion with 100% O_2_ without M101 supplementation appears unable to limit kidney fibrosis. Previous studies showed benefits of perfusion with 100% O_2_ versus none, but these studies were performed in different experimental conditions [3,8,28]. Expected results of two ongoing clinical trials from the Consortium for Organ Preservation in Europe (COPE), the first of which compares HMP + O_2_ between HMP in DCD kidneys (COMPARE Trial, ISRCTN32967929), and the second in ECD kidneys compares end-ischemic HMP + O_2_ or none (POMP Trial, ISRCTN63852508) will undoubtedly bring useful information [29,30].

Our results did not highlight a combined effect between our interventions, particularly in the early phase of recovery. This may be due to the opposing roles they play regarding oxidative stress.

While, in our hands, supplementation with O_2_ alone could be beneficial via tissue oxygenation, published data indicate that hyperoxia can induce ROS production and subsequent cell injury [4,21]. On the other hand, the intrinsic Cu/Zn-Superoxide dismutase (SOD) activity of M101 could limit IR-induced ROS toxicity [6]. Thus, the lack of additive effects of 100% O_2_ and M101 on kidney function, possibly stemmed from the ROS/anti-ROS balance being tipped in favor of ROS with an excess supply of O_2_ (100%) alone. On, the other hand, with M101 alone, the balance appears in favor of anti-ROS components.

Another hypothesis to explain this lack of additivity in the early phase would be that both strategies have the same target (bringing oxygen to the organ), but each one completes this task optimally. In this light, our conclusion that M101 offers a safer option to reach this objective is strengthened.

This could explain the better kidney function observed with M101 alone, at least in the early post-transplantation phase. Note however that we do observe the benefits of this combination on tissue lesion in the late phase (3 months). Dedicated experiments are warranted in order to dissect the various factors and contributions to the observed graft dynamic responses.

## 4. Materials and Methods

### 4.1. Experimental Model and Design of Experiments

Animal experiments were conducted at the MOPICT platform in Surgeres, France (This facility provides near-clinical conditions for both surgery and ICU-like follow up), in accordance with the French government and the Institutional Committee on the Ethics of Animal Experiments (France) (Accreditation number of the comity C2EA-84, approved protocol: CE2012-4, date of approval: December 2012). Animal studies conformed with ARRIVE guidelines.

For this study, we used 3-months-old male Large White pigs weighing ~40 kg. We designed an auto-transplant porcine model, mimicking injuries observed on kidney from DCD [10], in which the kidney undergoes 1 h of warm ischemia (WI) in situ by ligation of renal vessels before procurement and flush with 1L of cold PERF-GEN^®^ preservation solution (Institut Georges-Lopez, Lissieu, France). The organ is then perfused in the Waves^®^ machine perfusion (MP) (Waters Medical Systems LLC, Rochester, NY, USA and Institut Georges-Lopez) for 23 h at 4 °C in 1 L PERF-GEN^®^ solution at 40 mmHg perfusion. Finally, the kidney is re-implanted in the same animal after a nephrectomy of the contralateral kidney. We used an autotransplantation model to exclude the effects of the immunosuppressive drugs on the renal transplant, and to focus on ischemia-reperfusion (IR) effects. All operative parameters were identical between groups, and procedures were performed by same not awared operators. All surgical procedures were identical between groups and were performed by same operators in random conditions. The Waves^®^ hypothermic perfusion machine is a new MP, which could be connected to an external gas source, to diffuse the gas into the perfusion PERF-GEN^®^ solution, and thus to the kidney using the oxygenator membrane in the cassette and an air pump.

Four groups were studied (*n* = 6 per group) (Appendix A):

Group **W**: kidney perfused in Waves^®^ under normal room atmospheric conditions.

Group **W-O_2_**: kidney perfused in Waves^®^ connected to 100% O_2_ at 1 L/min (under hyperoxia atmosphere).

Group **W-M101**: kidney perfused in Waves^®^ under normal room atmosphere condition + 2 g/L M101 in PERF-GEN^®^ solution.

Group **W-O_2_ + M101**: kidney perfused in Waves^®^ connected to 100% O_2_ at 1 L/min (hyperoxia atmosphere) + 2 g/L M101 in PERF-GEN^®^ solution.

### 4.2. Ex Vivo Perfusion Parameters

Organ vascular resistance (mmHg/mL/min/g) and flow rate (mL/min) were measured in real time by the Waves^®^ software. We did not measure other parameters during ex vivo perfusion, such as O_2_ saturation, because the Waves^®^ perfusion machine was not adapted for sensor connection. In addition, sensors introduction into the perfusion kit would break the preservation procedure sterility.

### 4.3. Kidney Function and Biomarkers Evaluation

After kidney transplantation, the animals were put in an individual cage for one week. 24 h urine and blood samples were collected, from day 0 (normal value before transplantation) to day 90 post-transplantation, for creatinine, Aspartate transaminase (AST), Na^+^ and proteins determination, using a Cobas 8000 Modular-Analyzer (Roche-Diagnostic, Meylan, France) and for blood biomarkers measurement. 

Soluble Interleukine-18 (IL-18; Invitrogen Thermo Fisher Scientific (Illkirch, France), BMS672), Neutrophil Gelatinase-Associated-Lipocalin (NGAL: Eurobio (Courtaboeuf, France), 044) and Kidney-Injury-Molecule-1 (KIM-1-ectodomain; Eurobio, EP0102) were assessed in serum using ELISA kits according to the manufacturer’s instructions.

### 4.4. Renal Histochemical and Immunohistochemical Evaluation at 3 Months Post-Transplantation

Quantification of mast-cell infiltration in renal tissue was performed by immunohistochemistry using CD117 antibody (Abcam, Cambridge, UK). Quantification of vimentin positive cells was performed by immunohistochemistry using vimentin antibody (Sigma-Aldrich, Lyon, France). Histological evaluation of interstitial fibrosis by Sirius Red staining per field was quantified with ImageJ software. All histological evaluations were performed under blinded evaluation by a pathologist.

### 4.5. Statistical Analysis

Results are shown as a median with interquartile range (IQR). Statistical evaluations of kinetics are expressed in area under the curve (AUC) analysis, which is suitable since time is a continuous and dependent variable. All statistical analyses were performed with R Software using Kruskal-Wallis and Dunn’s post-test multiple comparison. A *p* value ≤0.05 was considered significant.

## 5. Conclusions

In conclusion, we showed that M101 supplementation during ex vivo preservation perfusion improved kidney graft recovery and outcome. Excess supply of oxygen did not improve outcome to the same level, and overall there was no additivity of effect between the strategies, especially in the early phase. However, the benefits of this combination on tissue lesions in the late phase suggest that an intermediate oxygenation level could be more efficient. Perfusion supplementation with M101 and O_2_ could also be valuable in other conditions, such as a sub-normothermic situation, which has been reported as being better than hypothermic perfusion in terms of organ preservation quality, as suggested by Morito et al [31]. Finally, these findings are potentially applicable to other organs, such as liver, for which MP techniques are just emerging.

## Figures and Tables

**Figure A1:**
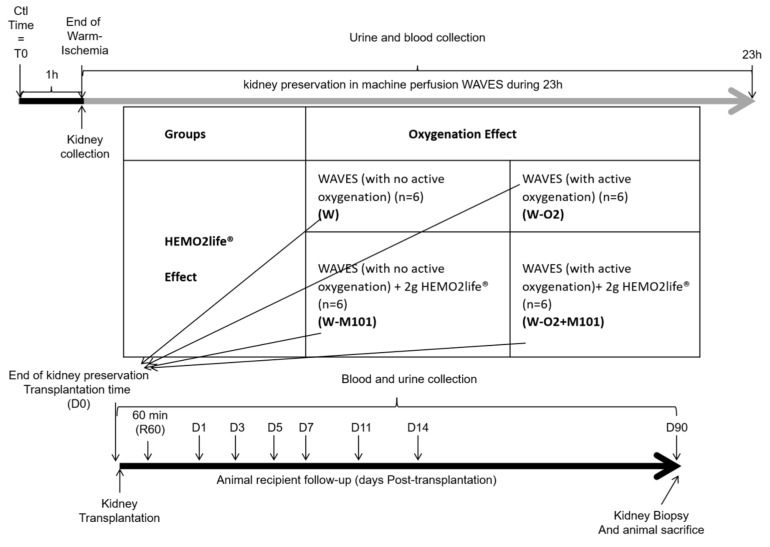
Experimental design.

## Abbreviations

AST	Aspartate transaminase
AUC	Area under the curve
DCD	Donation after circulatory death
DGF	Delayed graft function
ECD	Expanded criteria donors
FeNa	Fractional excretion of sodium
GFR	Glomerular filtration rate
IL-18	Interleukine-18
IR	Ischemia-reperfusion
IRI	Ischemia-reperfusion injuries
KIM-1	Kidney injury molecule-1
MP	Machine perfusion
NGAL	Neutrophil gelatinase-associated lipocalin
pmh	Per million habitants
SOD	Superoxide dismutase
